# What drives psychotherapists' willingness to treat individuals with spinal cord injury? A cross-sectional study from Germany

**DOI:** 10.3389/fpubh.2025.1704099

**Published:** 2026-01-13

**Authors:** Katja Oetinger, Anika Tyana Heudier, Alice Schewe, Katja Weimer, Yorck-Bernhard Kalke, Harald Gündel, Klaus Hönig

**Affiliations:** 1 Department of Psychosomatic Medicine and Psychotherapy, Ulm University Medical Centre, Ulm, Germany; 2 Department of Orthopaedics, Spinal Cord Injury Centre, Ulm University Medical Centre, Ulm, Germany

**Keywords:** disability-inclusive care, healthcare professionals' attitudes, mental health access barriers, mental health equity, outpatient psychotherapy, spinal cord injury

## Abstract

**Introduction:**

Individuals with spinal cord injury (SCI) have a higher prevalence of mental health problems than the general population but face significant barriers to accessing outpatient psychotherapy. Understanding the factors that influence therapists' willingness to treat this population is critical for improving mental healthcare equity.

**Methods:**

We conducted a cross-sectional online survey among licensed outpatient psychotherapists in Southern Germany. All therapists registered with the Association of Statutory Health Insurance Physicians who had an email address or an online contact form were invited to participate. In total, 677 complete datasets were analyzed in this study. Using logistic regression, we examined the associations between therapists' self-reported willingness to accept a hypothetical therapy request from an individual with SCI and nine potential influencing factors, including personal, emotional, and organizational variables.

**Results:**

Six variables were significantly associated with the therapists' willingness. Therapists who agreed to provide home-based therapy had higher odds of being in the willing group [OR = 2.28, 95% CI [1.50, 3.46], *p* < 0.001], as did those who reported a stronger feeling of preparedness [OR = 1.83, 95% CI [1.51, 2.21], *p* < 0.001] and greater field experience [OR = 1.34, 95% CI [1.11, 1.61], *p* = 0.002]. In contrast, older age [OR = 0.83, 95% CI [0.74, 0.92], *p* < 0.001], higher levels of emotional response [OR = 0.82, 95% CI [0.68, 0.99], *p* = 0.040], and workload concerns [OR = 0.73, 95% CI [0.55, 0.95], *p* = 0.020] were associated with lower odds of being in the willing group.

**Conclusion:**

Willingness to provide psychotherapy for individuals with SCI is associated with both modifiable (e.g., training, preparedness, home visit policies) and non-modifiable (e.g., age) factors. These findings highlight the importance of disability-specific education and structural adjustments to reduce access barriers. Although the present study was limited to Southern Germany, reports from other countries, such as Australia and Switzerland, document a low uptake of psychotherapy among individuals with SCI, suggesting that this may represent a broader challenge across healthcare systems. Our results thus contribute to a better understanding of provider-side barriers in Germany and may stimulate further international research into disability-inclusive mental healthcare provision.

## 1 Introduction

Although psychological care is a core component of comprehensive rehabilitation for individuals with spinal cord injury (SCI), there is mounting evidence suggesting a substantial under-provision of outpatient psychotherapy in various healthcare systems (1, 2). Previous research has documented patient-related and structural barriers to mental healthcare access for individuals with physical disabilities (3, 4). However, little is known about the role of outpatient psychotherapists in this process. In particular, it remains unclear how psychotherapists perceive individuals with SCI and the factors influencing their willingness to provide outpatient treatment. Addressing this gap requires a systematic examination of provider-related factors within the broader context of patient- and system-level barriers to care.

### 1.1 Background

SCI is a rare condition, affecting less than 0.1% of the global population (5), and similarly low prevalence rates have been reported in Germany (6). Despite its rarity, SCI is associated with profound long-term health consequences that extend far beyond mobility impairment alone. Individuals frequently experience secondary complications such as chronic pain, spasticity, bowel and bladder dysfunction, pressure ulcers, sexual dysfunction, and mental health problems (7–9). These complications often co-occur and require continuous management, contributing to substantial and enduring burdens (8, 9).

Mental health problems are particularly common among individuals with SCI. Compared to the general population, the prevalence rates of depression, anxiety, post-traumatic stress disorder, and chronic pain disorder are markedly higher (10–13). Psychological distress is particularly pronounced during transitional phases, such as the shift from inpatient rehabilitation to community living, often driven by challenges of social reintegration and perceived stigma (14–16). Secondary complications may persist or emerge long after the initial injury and can exacerbate psychological stress (17, 18). From a biopsychosocial perspective, physical and psychological processes are closely intertwined, underscoring the relevance of adequate psychotherapeutic support in the care of patients with SCI.

During inpatient rehabilitation, psychological care is typically integrated into specialized settings and has demonstrated efficacy in alleviating emotional distress (19). In contrast, the continuity of care after discharge is far less consistent. International observational studies have consistently reported low utilization of outpatient psychotherapeutic services among individuals with SCI. For instance, while approximately 80% of these individuals report visiting a general practitioner within a given year, only 8% in Switzerland and 12% in Australia report consulting a psychologist (1, 2).

Beyond the general context of SCI's impact, several patient-related factors also significantly contribute to the low utilization of outpatient psychotherapy among individuals with SCI. In the broader disability literature, stigma-related concerns have been frequently discussed. Fear of being perceived as “mentally ill” reduces help-seeking intentions, especially among those already facing stigma due to visible disabilities (20, 21). Evidence from SCI populations confirms that internalized stigma undermines psychosocial outcomes and engagement with psychological support (14, 15). Specifically, one study demonstrated that stigma mediates psychological adaptation (14), while another found associations with distress and lower community integration in the first year after discharge (15). Taken together, these findings suggest that stigma may represent an important patient-related mechanism contributing to the treatment gap in outpatient care. Beyond stigma, qualitative evidence suggests that anticipated barriers, such as limited physical accessibility of services and perceived inadequacies in therapists' disability-related expertise, may discourage service utilization among individuals with physical disabilities (22). Collectively, these individual-level factors represent important mechanisms contributing to the outpatient psychotherapy treatment gap. However, while highly relevant, such factors alone are unlikely to fully account for the pronounced underutilization observed, underscoring the need to consider system- and provider-related determinants of healthcare delivery.

Access to healthcare for individuals with SCI is also shaped by the structural characteristics of the healthcare system. International research consistently shows that people with mobility impairments encounter systemic barriers that restrict access to outpatient services, independent of individual help-seeking motivations (3, 4). The physical inaccessibility of healthcare facilities remains one of the most frequently reported obstacles, alongside organizational and financial barriers (3).

In outpatient settings, accessibility standards are often inconsistently applied. According to the German Federal Government's official participation report, only approximately 20% of medical practices meet core accessibility criteria, such as step-free access and wheelchair-accessible sanitary facilities (23). Complementing these national statistics, empirical studies in Germany have shown that individuals with SCI frequently report environmental barriers, such as inaccessible buildings and transportation difficulties, which are significantly associated with a reduced quality of life (24). Moreover, recent evidence from outpatient psychotherapists highlights that structural barriers to practice accessibility are common in routine outpatient care, underscoring the persistent difficulties in implementing legal accessibility standards in everyday care (25). In summary, the interplay between internalized stigma on the patient side and structural and environmental barriers on the system side creates a multifaceted barrier to care. Even when individuals with SCI overcome the psychological burden of help-seeking, they often face a healthcare infrastructure that is physically unprepared to meet their needs.

Within this context, provider-related factors may further contribute to the underutilization of outpatient psychotherapy by individuals with SCI. Psychotherapists act as key gatekeepers in outpatient mental healthcare, as access depends not only on physical accessibility but also on therapists' willingness, confidence, and perceived competence to treat individuals with complex physical disabilities. Limited disability-related knowledge and training have been identified as potential barriers. Psychotherapists typically receive little formal education on physical disabilities, resulting in gaps in their knowledge of disability-specific health issues, secondary complications, and the interaction between physical impairment and psychological processes (26, 27). This lack of knowledge fosters a sense of professional uncertainty. Therapists have reported concerns about making clinical mistakes, addressing medical issues beyond their expertise, or inadvertently causing harm due to insufficient understanding of physical limitations (25, 27). This uncertainty may manifest as avoidance behaviors, such as reluctance to accept patients with SCI.

Attitudinal factors and implicit biases may further influence clinical decision-making. As emphasized by Olkin (25), psychotherapists may be influenced by ableist norms and hold implicit assumptions about disabilities, dependence, or quality of life. Empirical evidence further shows that such assumptions can affect therapeutic expectations and willingness to engage in disability-related topics, even in the absence of explicit negative attitudes (28, 29). A recent meta-analysis demonstrated that implicit bias toward individuals with disabilities is both pervasive and robust across studies, underscoring its potential to hinder equitable therapeutic engagement (29).

A central theoretical framework for understanding these provider-related factors is Allport's contact hypothesis, which posits that meaningful interactions between social groups can reduce prejudice and improve intergroup attitudes (30). Subsequent research has demonstrated that positive, cooperative, and high-quality contact is particularly effective in reducing stigma and counteracting stereotypical assumptions (31). Recent disability-specific evidence suggests that engaging with positive exemplars can meaningfully reduce biased perceptions and foster more inclusive attitudes (32). In clinical contexts, such positive contact may foster greater openness, empathy and confidence when working with individuals with disabilities. Empirical evidence supports this notion: stigma toward disability can shape professional engagement and evaluative processes (14, 29), while disability-affirming identities and allyship have been identified as important factors in promoting more equitable and confident interactions with disabled individuals (33). Conversely, limited field experience may reinforce avoidance tendencies and reliance on stereotypes, particularly under the time and resource constraints of outpatient practice. Although much of this research addresses disability in general, these mechanisms are highly relevant to SCI, where therapists' confidence and willingness to engage are critical for ensuring access to appropriate psychological care.

### 1.2 Purpose and research question

While patient and structural barriers are well-documented, the role of provider-related factors, specifically psychotherapists' perceptions and willingness to treat individuals with SCI, remains critically underexplored. Addressing this specific lacuna, this study aimed to identify and examine the predictors of psychotherapists' willingness to treat individuals with SCI in outpatient settings, utilizing logistic regression to understand their relative contributions. These findings are essential for developing targeted interventions, guiding policy changes, and enhancing psychotherapeutic training to bridge the treatment gap for individuals with SCI.

Research Question: Which provider-related factors are associated with hypothetical outpatient psychotherapists' willingness to treat individuals with SCI?

## 2 Materials and methods

### 2.1 Design

This was a cross-sectional survey with prospectively collected data. This study was conducted and reported in accordance with the STROBE guidelines for cross-sectional studies. Data were collected between December 2020 and March 2021 using an online questionnaire. This study was approved by the Ethics Committee of the University of Ulm (approval number 337/20) and was conducted in accordance with the Declaration of Helsinki. Participants were recruited through professional mailing lists and psychotherapist networks. No incentives or compensation were provided, which reduced the likelihood of repeated participations. Active electronic informed consent was obtained prior to participation, and the participants were explicitly informed about the voluntary nature of the study, anonymous storage and analysis of their data, and exclusive use of data for scientific purposes. Data were collected and securely stored using Unipark survey software.

### 2.2 Participants

**Figure 1:**
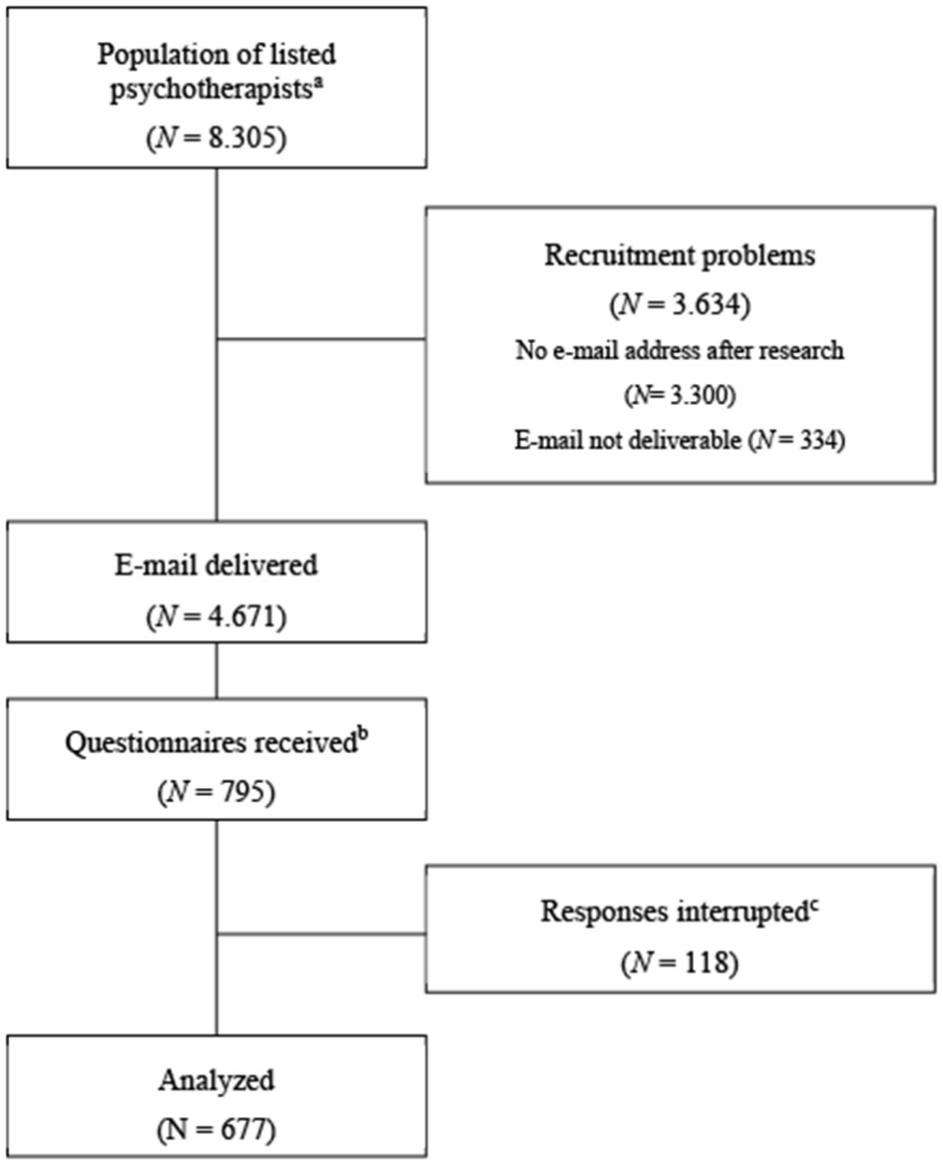
Participant flowchart. This study was aimed at listed psychotherapists in Southern Germany. ^a^Specialists in psychiatry were excluded. ^b^Returned questionnaires, whether fully or incompletely completed. ^c^Questionnaires were considered as “interrupted responses” if the outcome variable (therapists' willingness) remained unanswered.

This study focused on outpatient psychotherapists in Southern Germany listed by the Association of Statutory Health Insurance Physicians. At the time of the survey, 8.305 psychotherapists were listed with the Association of Statutory Health Insurance Physicians in the German states of interest. After intensive research on the contact details, 4.671 practices were successfully contacted via e-mail. Simultaneously, our study was advertised by umbrella organizations and professional associations on their websites. A total of 677 therapists completed the questionnaire and were included in the analysis. Figure 1 illustrates the flowchart from the population of interest to the final sample size. To minimize the risk of multiple submissions, no incentives or compensation were offered, making repeated participation improbable. As an additional safeguard, descriptive checks were performed using the background variables recorded by the survey platform (i.e., browser type and completion location). Cross-tabulation of these variables produced predominantly unique combinations of variables. Although duplicate participation cannot be ruled out entirely, the evidence indicates that this issue is unlikely to meaningfully affect the dataset.

### 2.3 Questionnaire development and variable overview

The questionnaire administered to therapists was developed in-house based on findings from prior studies addressing psychosocial care for minority groups, such as individuals with intellectual and physiological disabilities (34–36). To tailor the instrument to the specific context of SCI, additional items targeting perceived barriers and treatment-specific concerns were added (24).

#### 2.3.1 Demographic and therapeutic characteristics

General information on demographic and practice variables were obtained via self-report. This involved the variables *age* (selection across ten age categories), *sex* (“female,” “male,” or “diverse”) and *therapy approach* (no/yes each for all the therapy approaches approved in Germany: *Cognitive behavioral therapy, Analytical psychotherapy, Depth psychology-based psychotherapy* and *Systemic therapy*).

#### 2.3.2 Practice characteristics and other provision of therapy

Furthermore, the characteristics of the practice were surveyed. These included *location* (selection across the categories of rural municipality, small town, medium-sized city, and large city), average *waiting time* for a psychotherapy session (numerical query by week), and *accessibility*, which was divided into two stages. First, the general accessibility of the practice was surveyed (i.e., *perceived accessibility*). All respondents who affirmed accessibility in the first stage were shown the official accessibility criteria, such as no thresholds, extra-wide doors, and wheelchair-accessible toilets. Subsequently, the participants were asked to judge the accessibility of their practice again according to the official criteria (*official accessibility*). The therapists were also asked whether they could imagine conducting therapy sessions as part of *telepsychotherapy* (no/yes) or *home-based treatments* (no/yes).

#### 2.3.3 Treatment of patients with SCI

Information regarding further characteristics that may be related to the *therapist*s'* willingness* were then collected. The respective items asked about the therapists' *field experience* (“I already have experience in the psychotherapeutic treatment of SCI patients”), *emotional response* (“The thought of treating a psychosocially burdened patient with SCI and the associated severe physical limitations causes me to feel more personally impacted than other mental illnesses”) and the therapists' *feeling of preparation* (“I feel sufficiently prepared for working with SCI patients thanks to my psychotherapeutic training”) and were measured on a 5-point Likert scale (1 = *strongly disagree*, 5 = *strongly agree*).

#### 2.3.4 Concerns

Psychotherapists were asked whether they were concerned about *billing and diagnostic coding* (no/yes) or *scheduling problems* (no/yes) when treating patients with SCI.

Using a Likert scale (1 = *strongly disagree*, 5 = *strongly agree*), psychotherapists were asked whether they believed there would be an additional time or financial burden when working with patients with SCI in relation to the following aspects: (a) the indication for therapy over a particularly long period of time; (b) the involvement of caregivers and relatives; (c) special processing with regard to the past and altered future situation; (d) particularly close cooperation with practitioners from other fields; (e) difficulty in relationship building due to the presence of numerous shameful topics; and (f) other. These five items were averaged to the scale of *workload concerns*. The reliability analysis revealed an acceptable internal consistency with a Cronbach's alpha of 0.799 and good discriminatory power, with a corrected item-total correlation greater than 0.5 for each item.

#### 2.3.5 Need for information

Similarly, the scale *need for information* was formed as the average over 11 dichotomously surveyed items (no/yes), all of which asked about the desired need for information on various topics relating to life with SCI (e.g., bladder and bowel paralysis, dealing with sexuality, adapted sports, and leisure activities). The scale had excellent internal consistency, with a Cronbach's alpha of 0.924, and good discriminatory power, with an item-total-correlation greater than 0.5 for each item.

#### 2.3.6 Therapists' willingness

The outcome variable was *therapists' willingness* (“I could imagine treating a patient with SCI who needs psychotherapy”), which was measured on a 5-point Likert scale (1 = *strongly disagree*, 5 = *strongly agree*).

### 2.4 Statistical analyses

All analyses were performed using IBM SPSS Statistics, Version 29.0.2.0. For categorical variables, absolute and relative frequencies were reported. Continuous variables were presented as means, standard deviations, medians, and interquartile ranges. Cases with missing values in the relevant variables were excluded individually for univariate analyses so that all calculations were performed with all cases for which the necessary information was available. For the final regression analysis, listwise deletion was applied so that regressions were based on all cases with complete data in the identified variables. Missing data were minimal, with almost all items showing less than 2% missing data (range 0.15–6.35%). Thus, all items remained below the commonly accepted 10% threshold, with one exception: the dichotomous item on official accessibility. To address this, missing values for official accessibility were imputed with ‘no' under the assumption that if a practice was not perceived as generally accessible, it would also not meet official, more stringent criteria. All analyses were repeated. The results remained substantively unchanged, demonstrating that the findings were robust to our handling of missing data.

Non-response bias was evaluated by comparing the sample's demographic composition with the national statistics of psychotherapists in Germany (37). Sex and age distributions closely mirrored the national data. Moreover, the distribution of therapeutic orientations reflected the national allocation of psychotherapist seats across the four guideline-based procedures (38), with cognitive-behavioral therapy representing the largest share, followed by depth psychology-based psychotherapy and smaller proportions of analytic and systemic therapy. This consistent pattern supported the representativeness of the sample and reduced the likelihood of systematic non-response bias. Furthermore, potential early-late response bias was examined by comparing respondents from the first and second halves of the 3-month recruitment period. The groups showed comparable distributions in age, sex, and therapeutic orientation. As the invitations were distributed in a staggered and manual manner, formal statistical testing was not deemed methodologically appropriate. Nevertheless, the observed demographic similarity suggested a low risk of early–late response bias.

To assess the potential common method bias, Harman's single-factor test was conducted, including all Likert-scaled items. An unrotated principal component analysis revealed that the first factor accounted for 35.1% of the total variance, which is below the commonly accepted threshold of 50%.

The main goal of the analysis was to identify variables related to therapists' willingness. In the first step, bivariate relationships between the outcome variable on a five-point Likert scale and its possible predictor variables were calculated. Correlations between the outcome variable and all variables on a Likert scale were measured using Kendall's tau. The relationship between the outcome variable and dichotomous variables was calculated using the Mann–Whitney *U*-Test. Significant variables (*p* < 0.05; effect size of at least *r* = 0.1) were used as predictor variables for the second part of the analysis, the logistic regression.

To increase robustness and determine the unique effects of the variables, the analysis was repeated using binary logistic regression. For this purpose, the outcome variable was dichotomized for statistical reasons. The therapists were divided into a group of willing therapists (response options “strongly agree” and “tend to agree”) and a second group of doubting therapists (response options “partly agree/partly disagree,” “tend to disagree,” and “strongly disagree”). Logistic regression was performed to examine which variables contributed to outpatient therapists' willingness to accept a hypothetical therapy inquiry from a patient with SCI (0 = not willing, 1 = willing).

Variance inflation factor (VIF) was used to check for multicollinearity among the predictor variables. The relationship between each individual predictor and the outcome variable was assessed using odds ratios (OR). As those are controlled for in all other variables in the model, it ensures the robustness of the relationship. The proportion of variance explained in the outcome variable was computed using Nagelkerke *R*^2^.

## 3 Results

A total of 677 participants were included in this analysis. In relation to the 4,671 e-mails sent, this corresponds to a response rate of 14.5%. Relative to the population of all registered psychotherapists in Southern Germany, 8.2% of therapists were surveyed. Participant demographics are displayed in Table 1, and Table 2 presents the variables related to the treatment of SCI.

**Table 1:** Demographic characteristics.

**Variable**	**Distribution**			
	***n***	**%**	**M (SD)**	**Mdn (IQR)**
**Demographic and therapeutic characteristics**				
Age (*N* = 676)				
25–29 years	1	(0.1%)		
30–34 years	22	(3.3%)		
35–39 years	65	(9.6%)		
40–44 years	81	(12.0%)		
45–49 years	94	(13.9%)		
50–54 years	97	(14.3%)		
55–59 years	127	(18.8%)		
60–64 years	102	(15.1%)		
65 years or older	87	(12.9%)		
**Sex (*****N*** = **675)**				
Female	506	(75.0%)		
Male	169	(25.0%)		
Diverse	1	(0.1%)		
Cognitive behavioral therapy^a^	430	(63.5%)		
Analytical psychotherapy^a^	92	(13.6%)		
Depth psychology-based psychotherapy^a^	258	(38.1%)		
Systemic therapy^a^	38	(5.6%)		
**Practice characteristics**				
**Practice location**				
Rural municipality	66	(9.7%)		
Small town	166	(24.5%)		
Medium-sized city	182	(26.9%)		
Large city	263	(38.8%)		
Waiting time (weeks) (*N* = 650)			17.58 (15.08)	12.00 (16.00)

If not stated otherwise, *N* = 677. M, Mean; SD, Standard deviation; Mdn, Median; IQR, Interquartile range.

^a^Reflects the number and percentage of participants answering “yes” to this question.

**Table 2:** Variables on treatment of SCI.

**Variable**	**Distribution**			
	***n***	**%**	**M (SD)**	**Mdn (IQR)**
**Accessibility**				
Perceived accessibility^a^	208	(30.7%)		
Official accessibility^a^ (*N* = 577)	87	(15.1%)		
Home-based therapy^a^ (*N* = 635)	304	(44.9%)		
Telepsychotherapy^a^ (*N* = 666)	564	(83.3%)		
**Concerns**				
Concern about billing and diagnostic coding^a^	57	(9.5%)		
Concern about scheduling problems^a^	145	(24.2%)		
Workload concerns^b^			2.84 (0.86)	2.80 (1.20)
**Therapists' perceptions and experiences**				
Need for information^c^ (*N* = 674)			0.77 (0.31)	1.00 (0.41)
Field experience^d^ (*N* = 676)			1.83 (1.36)	1.00 (1.00)
Emotional response^d^ (*N* = 676)			2.55 (1.18)	2.00 (1.00)
Feeling of preparation^d^ (*N* = 676)			3.17 (1.15)	3.00 (2.00)
**Outcome variable**				
Therapists' Willingness^d^ (*N* = 676)				
Strongly disagree	29	(4.3%)		
Somewhat disagree	48	(7.1%)		
Partly agree/partly disagree	118	(17.5%)		
Somewhat agree	183	(27.1%)		
Strongly agree	298	(44.1%)		

If not stated otherwise, *N* = 677. M, Mean; SD, Standard deviation; Mdn, Median; IQR, Interquartile range.

^a^*Perceived accessibility* (“My practice is barrier-free and wheelchair accessible”), *Official accessibility* (“Could a patient with high-level paralysis using a wide electric wheelchair also access my practice without difficulty?”), *Home-based therapy* (“I could imagine offering sessions as part of outreach care”), *Telepsychotherapy* (“I could imagine conducting sessions via video call”), *Concern about billing and diagnostic coding* (“Do you have concerns regarding billing and diagnostic coding?”), *Concern about scheduling problems* (“Do you have concerns about scheduling issues such as short-notice cancellations or missed appointments?”) reflect the number and percentage of participants answering “yes” to each item (0 = *no*, 1 = *yes*).

^b^*Workload concerns* was computed as the average of five items rated on a 5-point Likert scale (1 = *strongly disagree*, 5 = *strongly agree*), assessing psychotherapists' perceived time or financial burden when treating patients with SCI across various aspects (“The indication for therapy over a particularly long period of time, the involvement of significant others and relatives, the special processing with regard to the past and changed future situation, the particularly close cooperation with practitioners from other fields of activity, the difficulty of building relationships due to the presence of numerous shameful topics, other aspects”).

^c^*Need for information* was measured as the average over eleven dichotomously surveyed items (0 = *no*, 1 = *yes*); (“When I imagine treating a patient with spinal cord injury, I would like to learn more about pain, pressure ulcers, spinal spasticity, bladder paralysis, rectal paralysis, sexual dysfunction, hoarseness and swallowing disorders, breathing problems depending on the level of paraplegia, coping with changes in self-image and body image, dealing with sexuality and intimacy, adapted sports and leisure activities”).

^d^*Field experience* (“I already have experience in the psychotherapeutic treatment of SCI patients”)*, Emotional Response* (“The thought of treating a psychosocially burdened patient with SCI and the associated severe physical limitations causes me to feel more personally impacted than other mental illnesses”)*, Feeling of Preparation* (“I feel sufficiently prepared for working with SCI patients thanks to my psychotherapeutic training”), *Therapists' Willingness* (“I could imagine treating a patient with SCI who needs psychotherapy”) were measured on a 5-point Likert scale (1 = *strongly disagree*, 5 = *strongly agree*).

Bivariate analyses were conducted to identify the variables related to the *therapists' willingness*. The 19 variables were selected as potential predictors. Within the bivariate analyses, differences in the variable *therapists' willingness* between the categories of dichotomous variables were tested via the Mann–Whitney *U*-Test (see Table 3 for an overview). For the gender variable, the diverse category was considered missing because of too few cases in the calculation.

**Table 3:** Comparison of categorical variables by therapists' willingness to treat patients with SCI.

**Variable**	***n***	***U***	***Z***	***p***	***r***
Sex^a^	674	39716.0	−1.43	0.152	0.055^c^
Cognitive behavioral therapy^b^	676	48620.0	−1.85	0.064	0.071^c^
Analytical psychotherapy^b^	676	24188.5	−1.49	0.137	−0.057^d^
Depth psychology-based psychotherapy^b^	676	49554.0	−1.85	0.065	−0.071^d^
Systemic therapy^b^	676	11549.0	−0.52	0.603	−0.020^d^
Home-based therapy^b^	634	36463.0	−6.31	**< 0.001** ^**^	0.251^c^
Telepsychotherapy^b^	665	22943.0	−3.43	**< 0.001** ^**^	0.133^c^
Perceived accessibility^b^	676	38089.5	−4.79	**< 0.001** ^**^	0.184^c^
Official accessibility^b^	576	17570.0	−2.76	**0.006** ^**^	0.115^c^
Concern about scheduling problems^b^	676	37247.0	−2.15	**0.032** ^*^	−0.083^d^
Concern about billing and diagnostic coding^b^	675	14387.0	−3.35	**< 0.001** ^**^	−0.129^d^

Mann–Whitney *U* tests were used for group comparisons. ^*^p < 0.05. ^**^p < 0.01.

^a^Female = 1, male = 2.

^b^Yes > no.

^c^Positive sign indicates a higher average rank of the higher category.

^d^Negative sign indicates a lower average rank of the higher category. Bold values indicate statistically significant results.

There was a significant result for six of the 11 potential categorical predictor variables. The rank of *therapists' willingness*, based on a 5-point Likert scale, was significantly higher with agreement to *home-based treatment* (*U* = 36463.0, *Z* = −6.31, *p* < 0.001), *telepsychotherapy* (*U* = 22943.0, *Z* = −3.43, *p* < 0.001), *perceived accessibility* (*U* = 3889.5, *Z* = −4.79, *p* < 0.001), and *official accessibility* (*U* = 17,570, *Z* = −2.76, *p* = 0.006). The rank of *therapists' willingness* was significantly lower with higher agreement to *concerns about scheduling problems* (*U* = 37247.0, Z = −2.15, *p* = 0.032), and *billing and diagnostic coding* (*U* = 14387.0, Z = −3.35, *p* < 0.001).

Four variables were considered for further logistic regression: *home-based treatment, telepsychotherapy, perceived accessibility*, and *concern about billing and diagnostic coding*. The variable *official accessibility* was excluded from further analysis due to its close logical dependence on *perceived accessibility* and notably smaller sample size. The effect size of the variable *concern about scheduling problems* was *r* = 0.084, which was lower than the predetermined cutoff of *r* = 0.1. Therefore, it was not considered further in the logistic regression.

For all at least ordinal-scaled variables, the bivariate correlation with *therapists‘ willingness* was calculated using Kendall's tau (Table 4). Six out of eight variables showed a significant correlation with the outcome variable: higher *age* (*r* = −0.131, *p* < 0.001), greater degree of *emotional response* (*r* = −0.216, *p* < 0.001), more *workload concerns* (*r* = −0.168, *p* < 0.001), and higher *need for information* (*r* = −0.084, *p* = 0.008) were associated with lower *willingness* scores. The higher the *field experience* (*r* = 0.204, *p* < 0.001) and the stronger the *feeling of preparation* (*r* = 0.348, *p* < 0.001), the higher the rank of *therapists' willingness*. The variable *need for information* showed a weak negative association with willingness (*r* = −0.084, *p* = 0.008) and was not included in the logistic regression.

**Table 4:** Correlations between predictor variables and therapists' willingness to treat patients with SCI.

**Variable**	***N***	***r***	**Sig. (2 tailed)**
Age	675	−0.131	**< 0.001** ^**^
Location	676	−0.013	0.701
Waiting time	649	0.050	0.101
Field experience	675	0.204	**< 0.001** ^**^
Emotional response	676	−0.216	**< 0.001** ^**^
Feeling of preparation	676	0.348	**< 0.001** ^**^
Workload concerns	676	−0.168	**< 0.001** ^**^
Need for information	673	−0.084	**0.008** ^**^

Correlations calculated using Kendall's τ. ^*^p < 0.05. ^**^p<*0.01*. Bold values indicate statistically significant results.

The final binomial logistic regression model used nine predictor variables to predict the dichotomized outcome variable of *therapists' willingness*. The dichotomized outcome variable comprised 481 willing (71.0%) and 195 doubtful (28.8%) therapists. The amount of variance explained by the binomial logistic regression model was represented by Nagelkerke's *R*^2^ = 0.309 and was statistically significant, χ^2^(9) = 150,48, *p* < 0,001. To control for multicollinearity, the VIF values were calculated for all model predictors. All values were below 1.3, indicating that multicollinearity was not a confounding factor in the analysis. All model coefficients and odds ratios are presented in Table 5.

**Table 5:** Factors associated with therapists' willingness.

**Variable**	**B**	**SE**	**Wald**	***p***	**Odds ratio**	**95% CI for odds ratio**	
						**LL**	**UL**
Age	−0.19	0.06	12.15	**< 0.001** ^**^	0.83	0.74	0.92
Home-based therapy	0.82	0.21	14.93	**< 0.001** ^**^	2.28	1.50	3.46
Telepsychotherapy	0.49	0.28	3.03	0.082	1.63	0.94	2.83
Accessibility practice	0.33	0.24	1.86	0.173	1.39	0.87	2.25
Concern about financial losses	−0.18	0.34	0.28	0.598	0.84	0.43	1.62
Field experience	0.29	0.09	9.54	**0.002** ^**^	1.34	1.11	1.61
Emotional response	−0.20	0.10	4.22	**0.040** ^*^	0.82	0.68	0.99
Workload concerns	−0.32	0.14	5.44	**0.020** ^*^	0.73	0.55	0.95
Feeling of preparation	0.60	0.10	38.36	**< 0.001** ^**^	1.83	1.51	2.21

Results are based on binary logistic regression with the outcome variable *therapists' willingness* (0 = not willing, 1 = willing). CI, confidence interval; LL, lower limit; UL, upper limit. ^*^p<*0.05*. ^**^p<*0.01*. Bold values indicate statistically significant results.

Of the nine variables entered into the regression model, six contributed significantly to predicting the *therapists' willingness*. Therapists who agreed with *home-based therapy* had higher odds of being in the willing group [OR = 2.28, 95% CI [1.50, 3.46], *p* < 0.001], as did those who reported a stronger *feeling of preparation* [OR = 1.83, 95% CI [1.51, 2.21], *p* < 0.001], and greater *field experience* [OR = 1.34, 95% CI [1.11, 1.61], *p* = 0.002].

In contrast, higher levels of *emotional response* [OR = 0.82, 95% CI [0.68, 0.99], *p* = 0.040], older *age* [OR = 0.83, 95% CI [0.74, 0.92], *p* < 0.001], and greater *workload concerns* [OR = 0.73, 95% CI [0.55, 0.95], *p* = 0.020] were associated with lower odds of being in the willing group. The other variables had no significant effects.

## 4 Discussion

This study investigated therapist-related factors associated with outpatient psychotherapists' willingness to accept hypothetical therapy inquiries from individuals with SCI. Due to the limited existing evidence, an exploratory approach was adopted. This study identified several significant associations. Willingness was most strongly associated with *openness to home-based therapy* (OR = 2.28), followed by stronger *feelings of preparation* (OR = 1.83) and greater *field experience* (OR = 1.34). In contrast, *older age* (OR = 0.83), heightened *emotional response* (OR = 0.82), and greater *workload concerns* (OR = 0.73) were associated with lower willingness.

The strongest association was observed for openness to home-based therapy. Although home-based psychotherapy is an established component of community mental health care in several countries, its implementation in patients with SCI has received little attention. Our findings suggest that flexible treatment settings may be more decisive than structural accessibility alone in shaping therapists' willingness to treat individuals with SCI. Although accessibility remains a prerequisite for equitable care, the therapists' openness in our sample was more strongly linked to their readiness to offer home-based therapy. Therefore, flexible service models may complement infrastructural accessibility by reducing the impact of mobility-related barriers (39). This finding builds upon previous research highlighting the scarcity of barrier-free psychotherapeutic practices in Germany (24) and aligns with the WHO health equity objectives (5), which emphasize that accessibility is a necessary but not sufficient condition for equitable healthcare.

Evidence from related populations supports this conclusion. International studies from the UK, USA, and Australia indicate that home-based psychotherapy can improve mental health outcomes, reduce inpatient treatment, and enhance quality of life (39–42). In the German context, a comparable and particularly instructive initiative is PSY-CARE (43), a structured program providing psychotherapy to community-dwelling older adults with depression and care needs. The project demonstrated that tailored home-based psychotherapeutic interventions are feasible within the German healthcare system and can lead to modest but clinically meaningful improvements in depressive symptoms. Although PSY-CARE has thus far been restricted to older adults, it illustrates that adapted service models can be implemented beyond inpatient psychiatry and within existing care structures. Taken together, these findings suggest that similarly structured approaches could be adapted for individuals with SCI, especially when mobility limitations or secondary health complications hinder regular outpatient attendance. More broadly, they underscore the potential of flexible, outreach-oriented psychotherapy models to bridge current accessibility gaps, highlighting the need for systemic support and targeted training to facilitate their implementation.

While flexible service models offer a crucial pathway to improved accessibility, our findings also highlight the equally vital role of therapists' internal resources. Specifically, their preparation and field experience were strongly associated with willingness, underscoring the importance of disability-specific expertise. Previous research has consistently shown that physical disability receives little attention in psychotherapeutic training (22, 26, 27), and many therapists report uncertainty when working with individuals with complex physical conditions (25, 44). Our findings further revealed gaps in awareness of accessibility standards, suggesting that openness alone does not translate into effective care if it is not accompanied by adequate preparation and training. Structured training and opportunities for supervised exposure to disability-related practice are therefore essential to transform willingness into competence and reduce mental healthcare stigmatization (25, 26).

Age was negatively associated with willingness, with younger therapists showing significantly greater willingness to treat patients with SCI. Although the underlying mechanisms remain unclear, this may reflect training-related cohort effects and exposure to evolving conceptual models. Frameworks such as posttraumatic growth (45) and positive disability identity (46), which emphasize resources and adaptation over traditional deficit-oriented perspectives, may be more deeply integrated into the curricula of younger cohorts. Thus, it remains to be seen whether our findings are explained by these educational changes or by the accumulation of (negative) professional experiences among older therapists over time. Future research should examine whether exposure to resource-oriented models mediates age-related differences in therapeutic openness.

A heightened emotional response was another factor associated with lower willingness to provide psychotherapy. When therapists feel overly emotionally affected, doubts about their professional competence and capacity to deliver adequate care may emerge as a form of self-protection. This finding aligns with previous research documenting emotional strain and uncertainty among psychotherapists working with people with disabilities (25) and highlights the need for self-awareness, disability-specific training, and structured supervision, as outlined in professional guidelines and empirical studies on competence development in psychology training (27, 35, 47). Importantly, such emotional reactions should not be understood solely as individual characteristics but also as potential indicators of limited experience and insufficient preparation for working with disability-related complexity.

Unreflected emotional responses may contribute to avoidance tendencies or subtle forms of stigmatization, particularly in outpatient settings characterized by time pressure and limited institutional support. As outlined in the introduction, Allport's contact hypothesis provides a useful framework for interpreting this pattern: meaningful, high-quality contact with members of stigmatized groups has been shown to reduce anxiety, counteract stereotypical assumptions, and foster more inclusive attitudes. From this perspective, heightened emotional reactivity may reflect a lack of prior positive contact experiences, which in turn reinforces uncertainty and deficit-oriented expectations.

Although contact processes were not directly assessed in the present study, disability-specific research suggests that engagement with peers with lived experience can meaningfully reduce biased perceptions and increase professional confidence (32). In the context of SCI, structured opportunities for contact, including peer-based formats, co-teaching initiatives, or supervised exchanges with individuals with SCI, may serve a dual function by improving patients' access to care and supporting therapists through normalizing disability, reducing emotional overload, and strengthening a disability-affirming professional stance.

Finally, workload concerns were negatively associated with the therapists' willingness. These concerns appear particularly realistic in the context of SCI, given the frequent comorbidities, secondary complications, and the need for multidisciplinary coordination (7–9). Previous research has shown that such organizational demands, including communication with multiple providers and short-notice cancellations, contribute to an increased workload and a higher risk of treatment discontinuity in outpatient psychotherapy (22, 25). These findings underscore the urgent need for structural and financial adjustments in outpatient mental healthcare, as current constraints are key determinants of restricted access (3, 4, 24).

In conclusion, our study offers preliminary insights into therapist-related factors that could inform future efforts to improve psychological care for people with SCI, paving the way for targeted interventions and policy reforms aimed at fostering a more inclusive and accessible mental healthcare system.

### 4.1 Implications for practice, education, and policy

Our findings necessitate a multilevel adaptation of the mental health care system to better serve the SCI population by addressing barriers at the provider, educational, and systemic levels of care.

#### 4.1.1 Clinical practice: enhancing service flexibility

To overcome the significant mobility and structural barriers inherent in SCI, service models must move beyond traditional clinic-based settings.

- Adaptive Delivery: Service models should adopt greater flexibility by integrating home-based therapy, telepsychotherapy, and online interventions as accessible treatment options. SCI centers should function as specialized outpatient hubs that coordinate flexible delivery modes to meet individual patient needs.
- Transitional Care: While not directly examined in this study, the identified structural barriers suggest that strengthening transitional support could mitigate care discontinuities during the transition from inpatient treatment to the home environment. To this end, SCI centers could implement ‘psychological bridge services' to facilitate timely follow-up and a seamless linkage to outpatient psychotherapy.

#### 4.1.2 Education: building disability-specific competence

Our findings indicate that general openness toward patients with SCI is insufficient without adequate preparation and field experience, underscoring the importance of disability-specific training.

- Integration of disability competence: There is an urgent need to integrate comprehensive disability-related knowledge, including physical disabilities, into both undergraduate psychology curricula and postgraduate psychotherapy training. Disability competence should be understood as a core element of mental health education rather than a niche topic tied to specific conditions.
- Experiential learning and contact: Consistent with the contact hypothesis, opportunities for structured interaction with individuals with disabilities and positive role models should be incorporated into training. Engaging with peers and positive role models is essential for trainees to develop a realistic, resource-oriented perspective and to recognize that a fulfilling life with a disability is possible.
- Supervision and professional support: When treating individuals with SCI, early access to supervision by clinicians with relevant field expertise should be strongly encouraged. Supervision can support self-reflection, help manage emotional responses, and address implicit biases, thereby promoting both therapist wellbeing and high-quality care.

#### 4.1.3 Policy and management: systemic alignment

For these practical and educational shifts to be sustainable, the underlying systemic and financial frameworks require adjustment.

- Support for interdisciplinary care: Management structures should facilitate effective interdisciplinary collaboration to mitigate workload concerns and organizational burden. Such support is essential when working with patients who present with complex physical and psychological comorbidities.
- Reimbursement Reform: Policymakers should consider reimbursement models that adequately cover flexible treatment settings, particularly home-based psychotherapy, as standard components of care for individuals with SCI rather than exceptional arrangements.
- Equity Standards: Achieving national and international health equity goals requires continuous monitoring and enforcement of accessibility standards. Collaboration between professional bodies, such as the Federal Chamber of Psychotherapists (BPtK), and patient organizations (e.g., the German Spinal Cord Injury Association, FGQ), may support the implementation of accessibility and inclusion standards within outpatient psychotherapy.

### 4.2 Limitations

#### 4.2.1 Methodological limitations

A primary limitation concerns the cross-sectional design and the absence of standardized instruments for assessing psychotherapists' attitudes toward SCI. The reliance on self-constructed items, while pragmatically necessary, constrains the interpretability of the findings and precludes causal inferences. Advancing this field will require the development and validation of standardized measures of disability-related competence, ideally complemented by multi-method approaches (e.g., combining self-report data with behavioral observations) to mitigate common method bias. Furthermore, given the sensitive nature of questions regarding willingness and attitudes toward a vulnerable population, social desirability bias might have influenced self-reported responses, potentially leading to an overestimation of willingness.

The study's representativeness is further limited by its recruitment strategy. The 14.5% response rate and exclusive reliance on digital mailing lists may have led to the underrepresentation of therapists who are less digitally active or professionally networked. As a result, the absolute levels of reported willingness should be interpreted cautiously. In contrast, associations between predictors and willingness are likely to be more robust against this form of selection bias, as this bias primarily affects prevalence estimates rather than relational patterns.

#### 4.2.2 Analytical and design limitations

Beyond these general methodological constraints, several limitations arise from specific design and analytical decisions. Most importantly, the willingness to provide psychotherapy was assessed using hypothetical scenarios. Although this approach was necessary given the low number of real-world therapy requests reported, it constitutes a central limitation for the study's substantive conclusions. Responses to hypothetical scenarios are likely to overestimate actual willingness, as real-life gatekeeping decisions are influenced by contextual pressures, such as workload, institutional constraints, and resource availability, which hypothetical judgments cannot fully capture. Consequently, the findings should be interpreted as reflecting attitudinal dispositions rather than enacted clinical behaviors. Future studies could address this intention–behavior gap by employing quasi-experimental designs, behavioral simulations, or vignette-based studies that systematically manipulate contextual constraints.

From an analytical perspective, dichotomization of the outcome variable to ensure sufficient statistical power may have obscured important nuances in therapists' responses. In particular, ambivalence or conditional acceptance cannot be adequately captured using a binary outcome. Studies with larger samples would benefit from preserving the ordinal structure of Likert-scale responses to enable more granular analyses.

#### 4.2.3 Scope limitations

Finally, the study is limited in scope by its exclusive focus on the provider perspective. Without incorporating the viewpoints of individuals with SCI, questions related to self-stigma, anticipated discrimination, and the actual success of help-seeking efforts remain unresolved. A more comprehensive understanding of access barriers would therefore require mixed-methods designs that integrate both supply- and demand-side perspectives and examine how therapists' willingness interacts with patient-level experiences and expectations.

## 5 Conclusion

The present study shows that *therapists' willingness* to accept hypothetical inquiries from individuals with SCI was associated with several variables, including *home-based therapy, feeling of preparation, field experience, age, emotional response*, and *workload concerns*. On the therapists' side, age represents a non-modifiable factor. In contrast, variables such as home-based treatment, field experience, feeling of preparation, and workload concerns appeared to be more adaptable through improved structural conditions. Emotional responses occupy an intermediate position: although they reflect personal attitudes and may be more resistant to change, they can still be shaped by training, awareness initiatives, and professional exposure. Our study suggests that future efforts could explore whether targeted support structures (e.g., supervision opportunities tailored to specific patient groups or compensation models for additional workload) may help reduce barriers. In conclusion, our study offers preliminary insights into therapist-related factors that could inform future efforts to improve psychological care for individuals with SCI.

## Data Availability

The raw data supporting the conclusions of this article will be made available by the authors, without undue reservation.
